# Experiences of schizophrenia patients with treatment buddy support during the COVID-19 pandemic

**DOI:** 10.4102/hsag.v29i0.2428

**Published:** 2024-05-10

**Authors:** Nomhle Mvunelo, Firoza Haffejee, Yasmeen Thandar

**Affiliations:** 1 Department of Basic Medical Sciences, Faculty of Health Sciences, Durban University of Technology, Durban, South Africa

**Keywords:** compliance, antipsychotic medication, medication adherence, schizophrenia, treatment support

## Abstract

**Background:**

Schizophrenia is a major psychiatric disorder affecting physical, psychosocial, and cognitive functioning. Treatment includes pharmacological and psychotherapeutic interventions. Adherence to prescribed medication is critical but reportedly low, because of side effects, failure to understand instructions, a lack of insight about the condition, cognitive deficits, and financial difficulties. Interventions to promote adherence to medication are required. This study introduced a treatment buddy to provide the patient with virtual support in adherence to medication.

**Aim:**

The aim of this study was to explore the participants’ lived experiences of a treatment buddy support.

**Setting:**

A specialised psychiatric clinic in a resource-constrained district of KwaZulu-Natal, South Africa.

**Methods:**

A qualitative study design, using semi-structured one-on-one interviews, was used to collect in-depth data from 24 participants, suffering from schizophrenia and who had been offered virtual treatment buddy support for 6 months. Data were analysed using thematic analysis.

**Results:**

The intervention improved adherence to medication. Participants indicated that the text messages served as reminders to take their medication daily. An alleviation of associated problems such as sleeping difficulties was observed. Participants were willing to encourage other patients suffering from schizophrenia to join ‘treatment buddy services’.

**Conclusion:**

The virtual treatment buddy support increased awareness of the importance to adhere to antipsychotic medications among patients suffering from schizophrenia and helped to resolve other schizophrenia-related problems experienced by the participants.

**Contribution:**

The study has provided a supportive intervention that can be utilised by mental health institutions to address poor adherence to medication by patients suffering from schizophrenia.

## Introduction

Schizophrenia is a major psychiatric disorder that causes psychosis affecting physical, psychosocial, and cognitive functioning (Patel et al. 2014). The disorder is characterised by several symptoms that include delusions, hallucinations, disorganised speech or behaviour, and impaired cognitive ability (Patel et al. 2014). It affects approximately 21 million people worldwide (Orrico-Sánchez et al. 2020).

Approaches towards care, treatment and rehabilitation of patients suffering from schizophrenia include pharmacological and psychotherapeutic interventions, with the former being regarded as more effective (Patel et al. 2014). There have been no significant advancements in the pharmacological treatment of psychosis in recent decades and the adverse effects of currently recommended treatment choices remain challenging (Harris 2023). Antipsychotic medications used to treat schizophrenia should be initiated immediately following an acute episode of schizophrenia before neurological changes, related to the disorder, occur in the brain (Patel et al. 2014). To achieve better treatment outcomes of antipsychotic medications, adherence to prescribed regimens is critical (Patel et al. 2014). Adherence to antipsychotics is generally low and ranges between 40% and 60% (García-Laredo 2018; Girma, Abdisa & Fikadu 2017; Tareke et al. 2018). A recent South African study reported that patients diagnosed with schizophrenia adhere to antipsychotic medication for only approximately 2 months, resulting in relapse of symptoms (Benelmokhtar et al. 2021). In contrast, Leijala et al. (2021) reported a high antipsychotic adherence rate of 75% in Finland. Adherence rates vary depending on patient circumstances, such as financial status, availability of family support, side effects, accessibility to mental health services, and improved cognitive functioning (Girma et al. 2017).

Low- and middle-income countries (LMICs), such as South Africa, have significant constraints regarding mental health human resource availability, infrastructure, and medication supply, all of which negatively impact the management of patients suffering from schizophrenia (Docrat et al. 2019). In South Africa, the healthcare system is characterised by a significant disparity, with two distinct tiers. The public sector, financed by the government, provides healthcare services to the majority, accounting for 71% of the population. In contrast, the private sector primarily relies on individual payments made to medical aid schemes or health insurance and serves approximately 27% of the population (Rensburg 2021). Public institutions are funded by government with a limited budget of which only 5% is utilised for mental health services (Docrat et al. 2019). The South African Society of Psychiatrists (SASOP) has observed that the allocated budget is declining because of budget cuts and raised concerns about the inadequate allocation of the national public health budget to mental healthcare in South Africa, despite a growing mental health crisis (SASOP 2020). The public sector therefore fails to provide the high-quality service necessary for its users. *The Mental Health Care Act (Act 17 of 2002)* caters for the provision of care, treatment and rehabilitation services to mental healthcare users and has provision for establishment of health review boards that ensure that mental health services are provided to all deserving individuals (Gazette 2002).

Several methods of improving treatment adherence have been previously investigated. Psychoeducation is a therapeutic approach in which clients are taught positive emotional and behavioural skills to improve life adjustment, management of emotions and self-awareness (Belmont 2020). Psychoeducation approaches are better implemented in the form of one-on-one individual counselling or a group therapy approach that involves groups of patients and support groups for their families (Belmont 2020). A combination of psychoeducational interventions together with drug optimisation is found to be more effective in improving adherence to treatment than psychoeducational approaches alone (Phan 2016). Motivational interviews have improved adherence among patients with schizophrenia (Dobber et al. 2018). The latter established a trusting relationship with the patients, who were influenced to develop a desire towards behaviour change and medication adherence. Fostering a therapeutic alliance, using reminder systems, and addressing substance use disorders have also been reported to improve adherence (Dobber et al. 2018). Positive therapeutic alliance that involves an ongoing collaboration between patient and therapist has proven to increase adherence (Arnow & Steidtmann 2014). Despite such provisions, non-adherence to psychiatric pharmacological treatment regimens remains a challenge in South Africa, thus warranting further investigation of interventions to improve adherence to antipsychotic medications.

A treatment buddy can have varying role responsibilities. Zuyderduin, Ehlers and van Der Wal (2008) refer to a treatment buddy as an individual, usually a trusted family member or friend who is committed to supporting a patient on taking their medications regularly. The treatment buddy may accompany the patient to the clinic and help them with taking their medication correctly, providing encouragement and supporting healthy practices directed towards an endeavour to recover (Zuyderduin et al. 2008). Treatment support involving the use of treatment buddies have been used successfully for patients with HIV and/or AIDS, cancer, and tuberculosis with the implementation of the Directly Observed Treatment Support programme (DOTS) (American Cancer Society 2021; Joseph et al. 2015; Nakamanya et al. 2018).

Treatment supporters to improve medication adherence in mental health have been investigated in previous studies, both globally (Andalibi & Flood 2021; Boardman, McCann & Kerr 2014; Duvivier & Lashmi 2021) and in South Africa (Mall et al. 2013; Naidoo, Gathiram & Schlebusch 2014; Sibeko et al. 2017). In an Australian study, peer support group intervention, which involved weekly phone calls over 2 months, improved treatment adherence, negative symptoms, and overall mental state in schizophrenic individuals. Peers were highly compliant individuals on maintenance treatment for their mental health (Boardman et al. 2014). In the United States of America (USA), more than 50 years ago, treatment buddies, which involved pairing of in-patients admitted for schizophrenia disorder with each other in the same ward, had positive outcomes (Ludwig & Arnold 1969). More recently in the USA, the participants of the Buddy Project, a movement aiming to prevent suicide by pairing people with buddies, were interviewed in a study with an aim to determine potential ways of designing a digital mental peer support system (Andalibi & Flood 2021). The study emphasised the significance of personalised and safe digital mental health peer support systems that prioritise matching users based on shared interests and identities (Andalibi & Flood 2021). A case report in Belgium by Duvivier and Lashmi (2021) recently reported the success of treatment buddy support provided to a migrant patient who suffered paranoid schizophrenia with several relapses post hospitalisation because of treatment non-adherence.

A South African study by Naidoo et al. (2014) reported the successful implementation of treatment buddies for suicidal patients in South Africa, with significant reduction in suicide attempts among participants who were part of the Buddy Intervention Support Group. A qualitative study in Cape Town which incorporated a treatment partner, and short message service (SMS) text messaging to support individuals with serious mental illness, including schizophrenia, in adherence to treatment reflected positive feedback (Mall et al. 2013). The treatment partners in the study were the mothers of the afflicted individuals with some participants documenting that they preferred a non-family member treatment partner. Also in Cape Town, Sibeko et al. (2017) used treatment-partner contracting, psychoeducation and sending of monthly text message reminders of clinic appointments to patients with severe mental disorders. They reported increased clinic visits, with improved adherence to medication resulting in greater symptom relief and improved quality of life, as well as a decrease in the relapse rate. The study, however, excluded patients with psychosis (Sibeko et al. 2017). Despite the successful implementation in many documented studies, the use of non-family treatment buddies to improve adherence to medication, in the context of schizophrenia, has not been documented in previous studies in South Africa.

This study introduced an intervention of a virtual treatment buddy support to assist with adherence to medication for patients suffering from schizophrenia by providing them with daily text message reminders for a duration of 6 months. Virtual treatment buddy support was used rather than physical support, in response to clinic regulations during the COVID-19 pandemic, to prevent overcrowding of the clinic and the spread of COVID-19.

The aim of this article is to present the participants’ lived experiences of this treatment buddy support.

## Research methods and design

### Study design

A qualitative approach utilising semi-structured one-on-one interviews was employed for this study. A questionnaire with six open-ended questions was developed by the researchers and utilised to guide the interview. The tool was pretested by an expert group, comprising of five academics.

### Study setting

This study was conducted in a specialised psychiatric clinic in Chatsworth, a resource-constrained district in eThekwini, KwaZulu-Natal, South Africa. This is a public sector clinic, which serves approximately 200 patients every month. Patients requiring hospitalisation are referred to a tertiary level hospital.

### Study population and sampling strategy

A non-probability, convenience sampling method was employed to recruit participants suffering from schizophrenia, who were over the age of 18 years and were prescribed pharmacologic treatment for a period of at least 6 months or more by a psychiatrist in the province of KwaZulu-Natal. Twenty-four participants, all of whom provided written informed consent, were recruited for this aspect of the study, which formed part of a larger research project. To ensure confidentiality and anonymity, no names were recorded during the interviews and the signed consent forms were kept in a separate folder from the interview transcripts. All participants were assigned participant numbers by which they were referred to.

Participants had to be fluent in either isiZulu and/or English and collect their monthly medication from the Chatsworth psychiatric clinic. Those with physical disabilities such as deafness and blindness as well as those who were intellectually challenged, were excluded.

### Intervention

The study was based on the understanding that adherence levels to medication can be more acceptable if patients were willing to take their medication as prescribed. A treatment buddy was allocated to each participant. A research assistant was appointed to act as a virtual treatment buddy for this study. Virtual treatment buddy support was used rather than physical support, in response to clinic regulations during the COVID-19 pandemic, to prevent overcrowding of the clinic and spreading the virus COVID-19. The treatment buddy sent daily text message reminders via WhatsApp to participants between 06:00 and 07:00 for the duration of the study period. Although a generic message was sent, these were sent individually to each participant, so that the research assistant could check whether the messages were read. The research assistant was a qualified psychiatric nurse and was therefore allowed to attend to telephonic queries from participants, conduct telephonic counselling, and attend to side effects of treatment reported to her by the participants.

### Data collection

All interviews were conducted post-intervention by the primary investigator (N.M.), who is fluent in both English and isiZulu, the two predominant languages in the province of KwaZulu-Natal. In-depth interviews were conducted in either of these languages, according to the preference of the participant. The following six key questions guided the interview process. If further explanations or clarifications were required, subsidiary questions were used to probe:

1. How do you feel about the treatment buddy support and the receipt of text message reminders to take medication over the past 6 months?
2. Explain how your treatment buddy helped you in taking your medication.
3. What other functions would you expect from a treatment buddy, besides sending reminders for you to take your medication? Please elaborate.
4. Describe any other assistance provided by the treatment buddy (not related to treatment).
5. Would you personally prefer to continue having a treatment buddy for yourself in future? Explain why.
6. Would you encourage other people suffering from schizophrenia to have treatment buddies for themselves? Explain why.

Each interview lasted approximately 30 min. Interviews were audio-recorded and safely stored until the data collection process was complete. The researcher transcribed the audio-recordings of all interviews. Because data saturation, where no new additional information was provided, was reached, more participants were not recruited.

### Data analysis

The data were analysed using Tesch’s eight steps (Datt & Chetty 2016), as follows: (1) The audio-recorded interviews were transcribed verbatim on a MicrosoftWord^®^ document. (2) The transcriptions were read, and notes were made on each script. All researchers independently familiarised themselves with the transcripts, where key points and relevant statements were highlighted. (3) The categories of primary information were utilised to generate codes. Creswell (2013) defines coding as the process of organising the material into ‘chunks’ before bringing meaning to it. (4) The coding process was used to generate themes, which were validated independently by two co-authors and verified by the third author. (5) The number of themes were reduced by grouping together similar topics. (6) Sub-categories that related to a particular theme were named as sub-themes. (7) Patterns were uncovered, and inferences drawn based on the generated themes; and (8) the data were interpreted. Triangulation discussions among researchers were used to verify all themes and to improve trustworthiness when reporting.

### Ethical considerations

Ethical clearance for this study was granted by Institutional Research Ethics Committee (IREC) of the Durban University of Technology (DUT) (IREC 030/20). Further permission was received from KwaZulu-Natal Department of Health (KZN DoH) (KZ_202103_008) and the Chief Executive Officer of R.K. Khan Hospital for use of Chatsworth Psychiatric Clinic. The study participants were aware that participation was voluntary, they could withdraw at any time without any penalty and that the information obtained would remain anonymous and confidentiality would be maintained. Written informed consent was obtained from those who agreed to participate, prior to them answering the questionnaire.

### Measures of trustworthiness

To ensure credibility, interviews were voice-recorded and subsequently transcribed. All transcriptions were verified by a second researcher. Information was probed during interviews until data were saturated and detailed notes were written immediately after the interview.

Consistency in the interviews was ensured by utilising the same interview guide for all applicants. The primary investigator coded the interviews; this was verified by all co-authors. The identified themes and subthemes were compared and any discrepancies in the data were discussed and modified until consistency was reached.

To ensure transferability of the results, a detailed description of the research setting and research processes has been provided.

## Results

The results first reflect the sociodemographic characteristics of the study, which is followed by a discussion on the themes and the relevant subthemes that emanated from the interviews.

The sociodemographic characteristics of the participants are detailed in Table 1. Most participants were male (63%; *n* = 15), of the Indian race (54%; *n* = 13), and unmarried (71%; *n* = 17). Just over half (54%; *n* = 13) possessed secondary education.

**TABLE 1:** Sociodemographic characteristics of participants (*N* = 24).

Sociodemographic characteristics	Frequencies	%
**Gender**		
Male	15	63
Female	9	37
**Age (years)**		
25–34	6	25
35–44	6	25
45–54	7	29
55–64	5	21
**Race**		
Black Africans	10	42
White people	1	4
Indians	13	54
**Language**		
IsiZulu	10	42
English	14	58
**Level of education**		
Primary	6	25
Secondary	13	54
Tertiary	4	17
Postgraduate	1	4
**Marital status**		
Single	17	71
Married	5	21
Widowed	2	8

**FIGURE 1:**
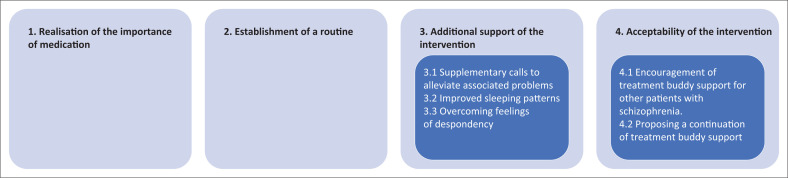
Themes and subthemes that emanated from the interviews.

Four key themes that emanated from the qualitative interviews included: realisation of the importance of medication, establishment of a routine, additional support of the intervention, and the acceptability of the intervention. The themes and related subthemes are indicated in Figure 1.

### Theme 1: Realisation of the importance of medication

The implementation of virtual support brought about the realisation of the importance of medication adherence. Having someone designated specifically to focus on the issue of medication, and the dedication of sending a reminder at almost the same time every day motivated the participants (P) and improved patient compliance:

‘I think they are good for me they remind me to take my medication when I forget, it motivates me to take my medication and helps me to live.’ (P3, Male, 35–44 years)

The participants realised that normal life functioning is dependent on the medication. Improvement was reported on self-care activities and relationships with family members and others, noted in the following responses:

‘It was very helpful to me because you don’t forget, the medication is actually carrying your life.’ (P10, Male, 35–44 years)

The following participant confirmed that the medication had a calming effect:

‘She sent messages every day. Helped me to be focused in taking my medication. Yes, it is very helpful. Makes the patient become more positive, taking medication will calm their mind.’ (P13, Female, 35–44 years)

The realisation that they could not get better without the medication was also manifested:

‘It helped me realize the importance of medication and the treatment I am receiving. Without medicines you cannot get better.’ (P58, Female, 65+ years)

### Theme 2: Establishment of a routine

Receiving daily messages from the treatment buddy led to patients developing a routine of taking medication at almost the same time every day, because the messages were sent to participants’ cell phones daily between 06:00 and 07:00:

‘It motivates us to always remember our treatment.’ (P10, Male, 35–44 years)

‘At least I’m getting up early on time, I take my medication on time. And I’m [o]k, and I’m taking as I was supposed to take in the morning and in the evening.’ (P79, Female, 55–64 years)

One participant explained her newly established routine following the cell phone bleep of the oncoming message:

‘When it rings, I wake up, it is an alarm to go have my tea and take my medication.’ (P102, Female, 45–54 years)

This was recapitulated by other participants:

‘When you get up in the morning and look at your WhatsApp messages and remember to take the medicines.’ (P17, Male, 45–54 years)

‘The message was fine. Every morning I know I must take my treatment.’ (P50, Female, 45–54 years)

The sending of messages by the research assistant was recognised by participants as a supplementary clinic service that created a sense of distinctive support for them:

‘She helped me to remember every day that when I’m taking tablets, I am not alone, there is someone helping me to remember.’ (P10, Male, 35–44 years)

### Theme 3: Additional support of the intervention

#### Subtheme 3.1: Supplementary calls to alleviate associated problems

The patients initially experienced many problems with the disorder and side effects of the medication. Among the problems that were reported by participants were loneliness and being suicidal. These could either be symptoms of schizophrenia or related to relapse and therefore proper management was crucial. The treatment buddy, who was a trained psychiatric nurse, used her knowledge to motivate the participants and reduce these problems. They were also able to control anger and improvement in fatigue and sleeping patterns were observed. Another participant reported that the daily treatment regimen alleviated the aggression problems, hence leading to better relationships:

‘Reminders helped me to take tablets so that everything comes right, and there is no more fighting.’ (P64, Female, 55–64 years)

When participants experienced problems they could call the treatment buddy for support, which was additional to the reminders that she was sending out daily:

‘Say for instance we have a problem; we can phone our treatment buddy to help us. The person is there for you if you have a problem. It’s good having someone you can talk to.’ (P17, Male, 45–54 years)

In addition, participants 1 and 34 reported that loneliness is also faced by schizophrenia patients, and they mentioned that they could call their treatment buddy during such times:

‘It was good, I expect assistance from the person I know, to talk to me because sometimes I am lonely.’ (P1, Male, 25–34 years)

Another participant mentioned that she gets suicidal at times and would appreciate having someone to talk to about her feelings, even if it is once a week:

‘Since I had a problem recently of attempting suicide, I would appreciate once a week a phone call.’ (P34, Female, 35–44 years)

In addition, some participants appreciated texting the treatment buddy about their personal problems. This created a communication system that afforded them confidentiality:

‘We had WhatsApp communication on the phone. Even today is not my appointment date but I talked to her.’ (P34, Female, 35–44 years)

Participant 13 shared the same sentiments when she suggested that more interaction was required as an extension of the treatment buddy system, such as hosting motivational discussions:

‘To have a motivational talk.’ (P13, Female, 45–54 years)

Participant 50 was escorted by her husband who reflected on how the medication improved his wife’s functioning. He confirmed that the additional empathetic support contributed to his wife’s coping:

‘I really appreciate the sisters from the clinic and from the hospital. My wife is in this situation because of them and I’m the only one who knows where we come from, I just want to say “Thank You” for the messages.’ (P50, Female, 45–54 years)

#### Subtheme 3.2: Improved sleeping patterns

Disturbance in sleeping patterns is a common problem among schizophrenic patients and is characterised by drowsiness. This could be as a result of a side effect of antipsychotic medication or a sign of relapse indicating that treatment is not taken correctly (Townsend 2015). Both disturbances in sleeping patterns are managed by a trained professional and can be prevented through regular medication intake. The role of a treatment buddy would be to monitor the occurrence and support the participant through motivation and referral.

Participants expressed that the treatment buddy helped them with enhanced physical functioning, which improved sleeping patterns:

‘It was good, beautiful, was good to get help. I was sleeping well.’ (P1, Male, 25–34 years)

Although other participants had their sleeping problems resolved, one participant still experienced disturbed sleeping patterns and requested further assistance:

‘My problem is not getting a good sleep, maybe if the buddy can help me with that.’ (P103, Male, 25–34 years)

#### Subtheme 3.3: Overcoming feelings of despondency

The feelings of despondency were alleviated by the support that the participants received. They realised that it would not be possible to overcome the disease without assistance:

‘The sickness I have is in the mind, if I don’t have support, how I will battle alone? If I battle alone the devil will win over me.’ (P3, Male, 25–34 years)

There was also a feeling that they would be rehabilitated after a few years:

‘Treatment buddy is always there for you, and in few years’ time we will all be secured.’ (P18, Male, 35–44 years)

One participant expressed her need for assistance regarding employment and was hopeful that engaging with the treatment buddy can improve her condition so that she can meaningfully look for employment. Unemployment is a substantial challenge among patients suffering from schizophrenia, resulting in frustration, despondency, and desperation:

‘I’m looking for a job, I need to support myself, but my illness makes it difficult for me to find the job.’ (P54, Male, 35–44 years)

### Theme 4: Acceptability of the intervention

#### Subtheme 4.1: Encouragement of treatment buddy support for other patients with schizophrenia

All participants agreed that they would encourage other patients who are suffering from schizophrenia to have treatment buddies. They were of the opinion that problems associated with the disorder such as psychotic episodes, failure to collect medications from the clinic, side effects and symptom relapse were resolved by the treatment buddy:

‘Yes, I would encourage them. I heard that other people stop taking medication, maybe they forgot to collect and then are afraid to go and collect the following day. Yes, I would encourage them. I see having a buddy as additional help for them.’ (P10, Male, 35–44 years)

Non-adherence leads to relapse and the participants felt that all patients would benefit from a treatment buddy service:

‘Yes, I would because it will remind them to take medication every day, and to avoid relapse.’ (P17, Male, 45–54 years)

Although participant 79 did not know of other patients with schizophrenia, he acknowledged that a treatment buddy would be useful to others and will also alleviate other problems:

‘I would encourage others, but I do not know anybody. If they get help, they will not be critical, worrying the family or disturbing them or making unnecessary noise.’ (P79, Female, 55–64 years)

Participant 19 was aware of another patient suffering from schizophrenia disorder and mentioned encouraging him to get a treatment buddy:

‘There is someone near home who has an illness of getting confused, I will encourage him.’ (P19, Male, 25–34 years)

One participant observed that other dysfunctional habits that are common among patients suffering from schizophrenia, such as substance abuse, can be eliminated when receiving treatment buddy support:

‘I would encourage others to get buddy support. If you’ve been smoking Zulu tobacco, you need to stop as it does not “gel” with this illness.’ (P103, Male, 25–34 years)

‘Yes, it is very helpful. Makes the patient become more positive, taking medication will calm their mind.’ (P13, Female, 45–54 years)

#### Subtheme 4.2: Proposing a continuation of treatment buddy support

When questioned about the willingness to continue with the treatment buddy support, all participants affirmed the suggestion of continuing with such support. They stated that reminders would enable the established routine to continue:

‘It motivates us to always remember our treatment.’ (P10, Male, 35–44 years)

‘I am happy in an amazing way when you’ve been sending messages because they were reminding me to take my medication.’ (P75, Female, 45–54 years)

‘I prefer to carry on, it truly helps.’ (P18, Male, 36–44 years)

Even those participants who had successfully established the routine of taking the medication and had become compliant prior to implementation of the treatment buddy support, appreciated the treatment buddy services:

‘Yes, it was nice to have a reminder although, I do not forget, I like them to continue coming.’ (P51, Female, 35–44 years)

They also affirmed that ongoing support would ameliorate the associated problems:

‘Yes, because it is helping to get everything out of the mind.’ (P1, Male, 25–34 years)

and

‘It is an additional support to getting medication. It is good.’ (P58, Female, 65+ years)

The satisfaction with the treatment buddy used in the study was also expressed:

‘I would prefer the messages and the same treatment buddy.’ (P17, Male, 45–54 years)

Participants reaffirmed their gratitude for the treatment buddy service. They could resume their normal daily functioning:

‘I would say thank you very much’ (P103, Male, 25–34 years)

‘Yes, it made me happy you know’ (P20, Female, 35–44 years).

In addition, participants viewed sending of messages as an additional service from the clinic and felt that it was sufficient:

‘The clinic is giving us more help; I think this is enough’ (P10, Male, 35–44 years).

Some participants also acknowledged the significance of the support they received from their relatives and friends. Those participants who received treatment buddy support, together with good support from family, friends and neighbours preferred a combined form of support:

‘Yes definitely, the same form of messages. I would prefer both messages and [*support from*] my people, family and friends.’ (P3, Male, 25–34 years)

‘It’s helping me, my sister is also reminding me to take [*medication*] every day.’ (P64, Female, 55–64 years)

## Discussion

Antipsychotic medication remains the cornerstone in the management of individuals who are diagnosed with schizophrenia disorder (Chien et al. 2013; Patel et al. 2014). Adherence to treatment is a critical step towards reducing relapse and improving symptomatology with improved emotional well-being and better treatment outcomes (El-Mallakh & Findlay 2015; Kane, Kishimoto & Correll 2013; Stentzel et al. 2018). The aim of this study was to implement a text message managed buddy support system to improve adherence to medication among patients diagnosed with schizophrenia.

This study revealed that the use of virtual treatment buddy support had a positive impact in assisting patients with schizophrenia to establish a routine in taking antipsychotic medication. The consistent daily reminders emphasised the significance of taking medication regularly, resulting in the patients realising its importance. Taking medication consistently at the same time every day is encouraged by therapists because it keeps the concentration of the drug within the therapeutic range and its therapeutic effect will not be compromised by missed doses (Duvivier & Lashmi 2021). Loots et al. (2021) reported that interventions involving motivation and medication self-management have produced positive outcomes regarding medication compliance.

The World Health Organization’s Mental Health Action Plan 2013–2020, the World Psychiatric Association, and the *Mental Health Care Act (Act 17 of 2002)* emphasised the need for community-based mental healthcare services (Gowda & Isaac 2022). The provisions of these bodies prescribe that patients with psychiatric disorders including those suffering from schizophrenia must be admitted for diagnosis and initiation of treatment, then get discharged to community-based integrated programmes. Community-based initiatives such as peer support groups and treatment support programmes can play a vital role in improving treatment adherence in schizophrenic patients who are not in hospital (Boardman et al. 2014). Peer support can be delivered in a variety of ways, including individual support, group support, and online support. In this study, the virtual treatment buddy support provided by the psychiatric nurse through daily text messaging where participants receive the text messages in their home environment, provides such community-based intervention intended to strengthen medication compliance. These demonstrated overall positive feedback and encouraged compliance among the study participants.

The impact of text messaging as a form of support for increasing adherence in mental health patients has been investigated in other studies. A South African qualitative study that assessed the feasibility of text messaging and treatment partner approach proposed that interventions comprise regular SMS reminders for medication intake and appointments (Mall et al. 2013). They recommended family members as not only suitable treatment partners for individuals with mental health issues but also allowing users to choose non-family partners. They also emphasised a collaborative, non-coercive approach with opportunities for social inclusion and broader social support networks, preventing family members from shouldering an undue care burden (Mall et al. 2013). Having a psychiatric nurse as the treatment buddy in our study ensured that undue burden was not posed on family members. A psychiatric nurse is also qualified enough to promote psychoeducation among the users, which was a well-received component among participants in a study by Sibeko et al. (2017).

Sibeko et al. (2017) implemented recommendations in a pilot randomised controlled trial, which consisted of treatment-partner contracting and psychoeducation, with monthly text message reminders of clinic appointments. They concluded that a treatment partner intervention is acceptable and feasible in an LMIC setting and had a positive impact on some secondary outcomes, such as quality of life and symptomatic relief. However, it is important to notice that the text messaging component of the intervention was not feasible in its current form. Among the reasons for this was that participants’ mobile numbers had changed and there was a loss of handsets, impacting negatively on the text messaging support (Sibeko et al. 2017). This study did not present with these issues. All participants received daily messages from their treatment buddy, in contrast to the monthly messages in the prior study, demonstrating that regular daily text messages yield better adherence.

This study, in contrast to previous research, offering various forms of treatment support, either digitally or in-person, highlighted the effectiveness of daily engagement by a single qualified nursing practitioner dedicated to contacting schizophrenic patients. This practitioner not only reminded patients to take their medication and attend clinic appointments but also possessed the expertise and knowledge required to provide psychoeducation when necessary. These focused efforts resulted in favourable outcomes.

Previous studies focused largely on peer group support. Andalibi and Flood (2021) explored digital peer support within the context of the Buddy Project, facilitated through digital means such as online communication platforms to support mental health patients. The authors interviewed users of the digital peer support system ‘Buddy Project’ and discovered that participants formed supportive friendships, which positively impacted their mental health. They also valued connecting with like-minded individuals. In this study, the text messages together with the involvement of the buddy was recognised by participants as an additional service from the clinic. It provided them with a feeling of being valued, as they recognised the presence of someone offering support. Furthermore, certain participants found solace in sharing their personal issues through text messages with the treatment buddy and conveyed their appreciation for this opportunity.

Patients suffering from schizophrenia have poor interpersonal relationships emanating from the inherent lack of trust because of the disorder (Pothimas et al. 2021). The lack of trust for both family members and healthcare workers, negatively impacts treatment adherence (Pothimas et al. 2021). In this study, some participants mentioned having private telephonic conversations with the treatment buddy and reported having benefited from the ability to have confidential discussions, illustrating the trusted alliance they established with the treatment buddy during the study. These confidential discussions particularly helped to alleviate loneliness. A participant who was suicidal mentioned that regular telephone calls, perhaps on a weekly basis would be preferred, so that these symptoms could be assuaged. Personal telephone calls would reassure patients; thus, a combination of text messages and verbal communication would be important in providing an opportunity for participants to clarify relevant matters with the treatment buddy. A similar finding was observed in another South African study in a primary care setting where the buddy intervention group was more effective than the control group in reducing suicidal behaviour. The control group witnessed three suicides, while the buddy group had one. Over the course of 18 months, there were a total of 171 additional suicide attempts, with 103 in the control group and 68 in the buddy group. These differences were statistically significant (Naidoo et al. 2014).

Peer support as a valuable addition to mental health services, benefits individuals with mental health issues by reducing hospitalisation, enhancing social functioning, improving quality of life, self-esteem, empowerment, and recovery. It can be delivered in various ways: individual, group, and online support. Implementing it presents challenges, including the need for well-trained and supervised peer support workers, and integration with other mental health services (Repper & Carter 2011). A recent systematic review found that group peer support interventions had a small but significant positive effect on the overall recovery in people with serious mental health conditions and suggested that peer support interventions may be a helpful adjunct to other treatments for people with mental health conditions (Lyons, Cooper & Lloyd-Evans 2021).

In this study, all the participants received pre-existing services from the clinic such as collecting their medication, assessments of mental state and physiological functioning, as well as referral to other services such as social workers or psychologists, when necessary. The treatment buddy support was a supplementary service that supported adherence to treatment. The participants applauded the benefits of the service.

The engagement of the treatment buddy with the current cohort of participants motivated the participants in performing activities of daily functioning. A lack of motivation in psychotic patients has been shown to impair goal-directed behaviours such as taking medication, which is necessary for effective daily life functioning and recovery (Favrod et al. 2019). Participants in this study further explained that the treatment buddy programme also helped to resolve other schizophrenia-related problems such as the inability to fall asleep. It has been reported that periods of non-adherence to antipsychotic medication have been linked to withdrawal symptoms as well as insomnia (Haddad, Brain & Scott 2014). Therefore, the regular reminders to ensure medication adherence helped to resolve the insomnia. Participants in this study reaffirmed the benefits of a routine schedule because they often experienced cognitive impairments such as memory loss, more so with information that was communicated verbally. The text message is therefore most suitable because they could read their messages, without relying on verbal instructions.

Participants suggested that this service of buddy support with daily text messaging reminders be extended to other patients suffering from schizophrenia as it would benefit others with the condition. In addition, there was a willingness for the continuation of the service.

### Limitations

Patients were recruited from a single clinic in KwaZulu-Natal only, hence the results cannot be generalised to other regions of the country. This study was a qualitative exploration of the patients’ views of the treatment buddy support and did not measure actual compliance, which would be important in determining whether uptake of medication improved.

### Recommendations

Considering the criticality of adherence to antipsychotic medications and the positive response from participants of this study, the provision of treatment buddies by all mental health institutions is recommended for future psychiatric care. Future studies should quantify actual adherence before and after such an intervention.

## Conclusion

The virtual treatment buddy support increased awareness of the importance of adherence to antipsychotic medications among patients suffering from schizophrenia and helped to resolve other schizophrenia-related problems, such as insomnia, that were experienced by the participants.
